# Correction: Self-assembly of bovine serum albumin (BSA)–dextran bio-nanoconjugate: structural, antioxidant and *in vitro* wound healing studies

**DOI:** 10.1039/d1ra90078a

**Published:** 2021-02-08

**Authors:** Sonali S. Rohiwal, Z. Ellederova, Arpita P. Tiwari, Mohammed Alqarni, Sara T. Elazab, Gaber El-Saber Batiha, Shivaji H. Pawar, Nanasaheb D. Thorat

**Affiliations:** Centre for Interdisciplinary Research, D. Y. Patil University Kolhapur – 416006 MH India; The PIGMOD Center, Institute of Animal Physiology and Genetics, v. v. i., The Czech Academy of Sciences Libechov Czech Republic; Department of Pharmaceutical Chemistry, College of Pharmacy, Taif University P.O. Box 11099 Taif 21944 Saudi Arabia; Department of Pharmacology, Faculty of Veterinary Medicine, Mansoura University Mansoura 35516 Egypt; Centre for Innovative and Applied Research, Anekant Education Society, T. C. College Campus Baramati MH India; Division of Medical Science, Nuffield Department of Women’s & Reproductive Health, University of Oxford Oxford UK thoratnd@gmail.com; Department of Stem Cell and Regenerative Medicine, D. Y. Patil Education Society, Deemed to Be University Kolhapur India; Department of Pharmacology and Therapeutics, Faculty of Veterinary Medicine, Damanhour University Damanhour 22511 Al Beheira Egypt

## Abstract

Correction for ‘Self-assembly of bovine serum albumin (BSA)–dextran bio-nanoconjugate: structural, antioxidant and *in vitro* wound healing studies’ by Sonali S. Rohiwal *et al.*, *RSC Adv.*, 2021, **11**, 4308–4317, DOI: 10.1039/D0RA09301G.

The authors regret that the affiliations for Gaber El-Saber Batiha were incorrectly shown in the original manuscript. The corrected list of affiliations is as shown above.

The Royal Society of Chemistry apologises for these errors and any consequent inconvenience to authors and readers.

## Supplementary Material

